# 3-(4-Chloro­phen­yl)-2,1-benzisoxazole-5-carbonyl chloride

**DOI:** 10.1107/S1600536808037872

**Published:** 2008-11-22

**Authors:** Yuriy Teslenko, Vasyl Matiychuk, Mykola Obushak, Vasyl Kinzhybalo, Katarzyna Ślepokura

**Affiliations:** a Department of Organic Chemistry, Ivan Franko National University of Lviv, Kyryla and Mefodiya 6, Lviv, 79005, Ukraine; b Faculty of Chemistry, University of Wrocław, 14 Joliot-Curie St, 50-383 Wrocław, Poland

## Abstract

The molecule of the title compound, C_14_H_7_Cl_2_NO_2_, is not planar; the dihedral angle between the mean planes of the chloro­phenyl and benzisoxazole rings is 20.32 (7)°. The carbonyl chloride group is twisted with respect to the benzisoxazole ring by 2.5 (1)°. The mol­ecular conformation is stabilized by an intra­molecular C—H⋯Cl hydrogen bond. In the crystal packing, adjacent mol­ecules are linked into dimers by inter­molecular C—H⋯O hydrogen bonds. The dimers are further stacked into columns along the unique axis direction by π–π stacking inter­actions, with a centroid⋯centroid distance of 3.828 (5) Å. Other weak inter­molecular C—H⋯O and C—H⋯Cl inter­actions are also present.

## Related literature

For the applications and biological activities of benzo[*c*]isoxazoles, see: McEvoy *et al.* (1968 ▶); Hester *et al.* (1989 ▶); Walsh *et al.* (1990 ▶); Angibaud *et al.* (2003 ▶). For details of the synthesis, see: Davis & Pizzini (1960 ▶). For hydrogen-bond motifs, see: Bernstein *et al.* (1995 ▶).
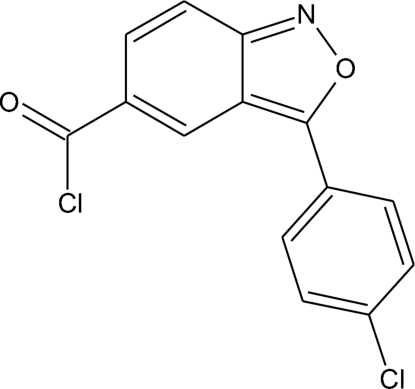


## Experimental

### 

#### Crystal data

C_14_H_7_Cl_2_NO_2_
*M* *_r_* = 292.11
Monoclinic, 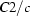

*a* = 30.337 (6) Å
*b* = 3.828 (1) Å
*c* = 21.000 (4) Å
β = 100.67 (3)°
*V* = 2396.6 (9) Å^3^
*Z* = 8
Cu *K*α radiation
μ = 4.85 mm^−1^
*T* = 100 (2) K
0.80 × 0.15 × 0.12 mm

#### Data collection

Oxford Xcalibur PX κ-geometry diffractometer with Onyx CCD camera
Absorption correction: analytical (**CrysAlis RED**; Oxford Diffraction, 2006 ▶) *T*_min_ = 0.21, *T*_max_ = 0.66
9609 measured reflections
2413 independent reflections
2131 reflections with *I* > 2σ(*I*)
*R*_int_ = 0.068

#### Refinement

*R*[*F* ^2^ > 2σ(*F* ^2^)] = 0.044
*wR*(*F* ^2^) = 0.126
*S* = 1.05
2413 reflections
173 parameters
H-atom parameters constrained
Δρ_max_ = 0.30 e Å^−3^
Δρ_min_ = −0.60 e Å^−3^

### 

Data collection: *CrysAlis CCD* (Oxford Diffraction, 2006 ▶); cell refinement: *CrysAlis RED* (Oxford Diffraction, 2006 ▶); data reduction: *CrysAlis RED*; program(s) used to solve structure: *SHELXS97* (Sheldrick, 2008 ▶); program(s) used to refine structure: *SHELXL97* (Sheldrick, 2008 ▶); molecular graphics: *DIAMOND* (Brandenburg, 2005 ▶); software used to prepare material for publication: *publCIF* (Westrip, 2008 ▶).

## Supplementary Material

Crystal structure: contains datablocks I, New_Global_Publ_Block. DOI: 10.1107/S1600536808037872/rz2265sup1.cif

Structure factors: contains datablocks I. DOI: 10.1107/S1600536808037872/rz2265Isup2.hkl

Additional supplementary materials:  crystallographic information; 3D view; checkCIF report

## Figures and Tables

**Table 1:** Hydrogen-bond geometry (Å, °)

*D*—H⋯*A*	*D*—H	H⋯*A*	*D*⋯*A*	*D*—H⋯*A*
C4—H4⋯Cl1	0.95	2.58	3.015 (2)	108
C6—H6⋯O1^i^	0.95	2.53	3.378 (2)	149
C9—H9⋯Cl1^ii^	0.95	2.96	3.813 (2)	150
C13—H13⋯O2^iii^	0.95	2.69	3.492 (2)	142

Symmetry codes: (i) 
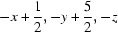
; (ii) 
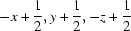
; (iii) 
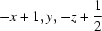
.

**Fig. 1.**
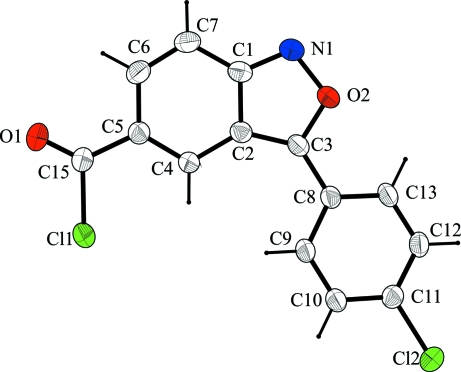
The molecular structure of the title compound with the atom labeling scheme. Displacement ellipsoids are drawn at the 50% probability level.

**Fig. 2.**
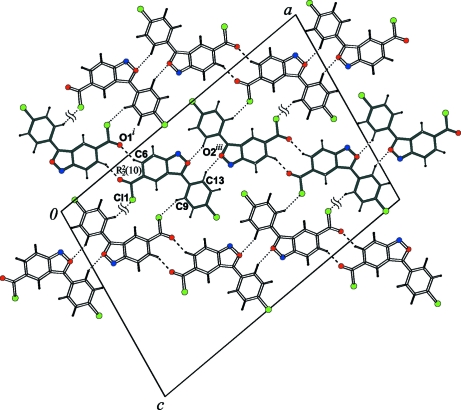
Packing diagram of the title compound viewed along the b axis, showing intermolecular hydrogen bonds (dashed lines) and weak hydrogen interactions (dotted lines). Symmetry codes are as in Table 1.

## supplementary crystallographic information

### Comment

Our interest in benzo[*c*]isoxazoles is concerned with their biological activity and their application as precursors of the variety of bioactive compounds (Angibaud *et al.*, 2003; Walsh *et al.*, 1990; Hester *et al.*, 1989; McEvoy *et al.*, 1968). The title compound will be used in our further investigations as a building block for modifications by nucleophiles.

The asymmetric unit of the title compound is shown in Figure 1. The planarity of the molecule is disturbed by intramolecular attractive and repulsive interactions of the C–H···Cl, C–H···O and C–H···H–C types. The dihedral angle between the least-square mean planes of the chlorophenyl and benzisoxazole rings is 20.32 (7)°. The carbonyl chloride group is rotated by 2.5 (1)° with respect to the benzisoxazole ring. An intramolecular C—H···Cl hydrogen bonding interactions is observed (Table 1). The crystal packing (Fig. 2) is governed by intermolecular interactions of the C–H..O type and by π-π stacking interactions. The C–H···O type hydrogen bonds connect adjacent molecules into dimers, forming ten-membered rings of graph set motif *R*^2^_2_(10) (Bernstein *et al.*, 1995). The dimers are further linked along the unique axis direction by π-π stacking interactions: *Cg1*···*Cg1*^i^ = *Cg2*···*Cg2*^i^ = 3.828 (5) Å (*Cg1* and *Cg2* are the centroids of the C1/C2/C4–C7 and C8–C13 aromatic rings, respectively; symmetry code: (i) x, 1+y, z); the corresponding perpendicular interplanar distances are 3.429 (4) and 3.475 (4) Å, respectively, and the centroid-centroid offsets are 1.702 (4) and 1.605 (3) Å, respectively. Additionally, two weak C–H···O and C—H···Cl interactions are present in the structure (shown as dotted lines in Figure 2).

### Experimental

To 40 ml of an ethanol solution of potassium hydroxide (4 g, 0.1 mol) *p*-nitrobenzoic acid (1.67 g, 10 mmol) was added with stirring. Then 5 ml of an ethanol solution of 4-chlorophenylacetonitrile (1.82 g, 12 mmol) was added to the reaction mixture. The suspension was stirred for 4 h at 323 K and left overnight at room temperature. The reaction mixture was poured into 150 ml of water and acidified with hydrochloric acid. The precipitate was isolated by filtration, washed with water and dried. Crude acid was added to a solution of thionyl chloride (1.19 ml, 20 mmol) in benzene (15 ml) and heated under reflux until a clear solution was obtained. Yellow needles of the title compound were obtained by slow cooling of the reaction solution (m.p. 453–454 K; 2 g, yield 70%).

### Refinement

All H atoms were found in difference-Fourier maps. In the final refinement cycles, H atoms were positioned geometrically and treated as riding atoms, with C–H = 0.95 Å and with *U*_iso_(H) = 1.2 *U*_eq_(C).

### Crystal data

**Table:**

C_14_H_7_Cl_2_NO_2_	*F*_000_ = 1184
*M**_r_* = 292.11	*D*_x_ = 1.619 Mg m^−^^3^
Monoclinic, *C*2/*c*	Melting point: 453-454 K K
Hall symbol: -C 2yc	Cu *K*α radiation λ = 1.54180 Å
*a* = 30.337 (6) Å	Cell parameters from 9042 reflections
*b* = 3.8280 (10) Å	θ = 2.2–76.9º
*c* = 21.000 (4) Å	µ = 4.85 mm^−^^1^
β = 100.67 (3)º	*T* = 100 (2) K
*V* = 2396.6 (9) Å^3^	Needle, yellow
*Z* = 8	0.80 × 0.15 × 0.12 mm

### Data collection

**Table:**

Xcalibur PX κ-geometry diffractometer with CCD Onyx camera	2413 independent reflections
Radiation source: fine-focus sealed tube	2131 reflections with *I* > 2σ(*I*)
Monochromator: graphite	*R*_int_ = 0.068
*T* = 100(2) K	θ_max_ = 76.7º
ω and φ scans	θ_min_ = 4.3º
Absorption correction: analytical(*CrysAlis RED*; Oxford Diffraction, 2006)	*h* = −38→21
*T*_min_ = 0.21, *T*_max_ = 0.66	*k* = −4→4
9609 measured reflections	*l* = −20→26

### Refinement

**Table:**

Refinement on *F*^2^	Hydrogen site location: inferred from neighbouring sites
Least-squares matrix: full	H-atom parameters constrained
*R*[*F*^2^ > 2σ(*F*^2^)] = 0.044	*w* = 1/[σ^2^(*F*_o_^2^) + (0.1002*P*)^2^] where *P* = (*F*_o_^2^ + 2*F*_c_^2^)/3
*wR*(*F*^2^) = 0.126	(Δ/σ)_max_ = 0.001
*S* = 1.05	Δρ_max_ = 0.30 e Å^−^^3^
2413 reflections	Δρ_min_ = −0.60 e Å^−^^3^
173 parameters	Extinction correction: SHELXL, Fc^*^=kFc[1+0.001xFc^2^λ^3^/sin(2θ)]^-1/4^
Primary atom site location: structure-invariant direct methods	Extinction coefficient: 0.00084 (17)
Secondary atom site location: difference Fourier map	

### Special details

**Table:**

Geometry. All e.s.d.'s (except the e.s.d. in the dihedral angle between two l.s. planes) are estimated using the full covariance matrix. The cell e.s.d.'s are taken into account individually in the estimation of e.s.d.'s in distances, angles and torsion angles; correlations between e.s.d.'s in cell parameters are only used when they are defined by crystal symmetry. An approximate (isotropic) treatment of cell e.s.d.'s is used for estimating e.s.d.'s involving l.s. planes.
Refinement. Refinement of *F*^2^ against ALL reflections. The weighted *R*-factor *wR* and goodness of fit *S* are based on *F*^2^, conventional *R*-factors *R* are based on *F*, with *F* set to zero for negative *F*^2^. The threshold expression of *F*^2^ > σ(*F*^2^) is used only for calculating *R*-factors(gt) *etc*. and is not relevant to the choice of reflections for refinement. *R*-factors based on *F*^2^ are statistically about twice as large as those based on *F*, and *R*- factors based on ALL data will be even larger.

### Fractional atomic coordinates and isotropic or equivalent isotropic displacement parameters (Å^2^)

**Table:**

	*x*	*y*	*z*	*U*_iso_*/*U*_eq_	
Cl1	0.226537 (14)	0.66461 (14)	0.17672 (2)	0.0394 (2)	
Cl2	0.440884 (15)	0.22145 (13)	0.50018 (2)	0.0379 (2)	
O2	0.44488 (4)	0.9282 (4)	0.21086 (6)	0.0344 (3)	
O1	0.22228 (5)	0.9426 (5)	0.06384 (7)	0.0531 (4)	
N1	0.43252 (5)	1.0756 (5)	0.14821 (8)	0.0373 (4)	
C3	0.40860 (5)	0.8178 (5)	0.23358 (8)	0.0276 (4)	
C1	0.38838 (6)	1.0480 (5)	0.13517 (8)	0.0305 (4)	
C2	0.37105 (5)	0.8870 (5)	0.18709 (8)	0.0272 (4)	
C7	0.35923 (6)	1.1558 (5)	0.07744 (9)	0.0348 (4)	
H7	0.3706	1.2660	0.0433	0.042*	
C6	0.31470 (6)	1.0960 (5)	0.07275 (8)	0.0341 (4)	
H6	0.2946	1.1668	0.0347	0.041*	
C5	0.29704 (5)	0.9272 (5)	0.12426 (8)	0.0286 (4)	
C4	0.32426 (5)	0.8258 (5)	0.18041 (8)	0.0277 (4)	
H4	0.3122	0.7171	0.2141	0.033*	
C8	0.41646 (5)	0.6636 (5)	0.29788 (8)	0.0277 (4)	
C9	0.38161 (5)	0.6433 (5)	0.33305 (8)	0.0312 (4)	
H9	0.3526	0.7261	0.3143	0.037*	
C10	0.38894 (5)	0.5044 (5)	0.39469 (8)	0.0325 (4)	
H10	0.3652	0.4905	0.4183	0.039*	
C11	0.43141 (6)	0.3854 (5)	0.42168 (8)	0.0297 (4)	
C12	0.46655 (5)	0.3991 (5)	0.38806 (8)	0.0316 (4)	
H12	0.4954	0.3148	0.4072	0.038*	
C13	0.45895 (5)	0.5378 (5)	0.32618 (8)	0.0314 (4)	
H13	0.4828	0.5478	0.3026	0.038*	
C15	0.24813 (6)	0.8697 (5)	0.11157 (9)	0.0338 (4)	

### Atomic displacement parameters (Å^2^)

**Table:**

	*U*^11^	*U*^22^	*U*^33^	*U*^12^	*U*^13^	*U*^23^
Cl1	0.0264 (2)	0.0502 (4)	0.0408 (3)	−0.00458 (17)	0.00416 (19)	0.00756 (19)
Cl2	0.0379 (3)	0.0451 (3)	0.0291 (3)	0.00141 (18)	0.00193 (19)	0.00423 (17)
O2	0.0246 (6)	0.0479 (8)	0.0318 (6)	−0.0032 (5)	0.0085 (5)	0.0005 (5)
O1	0.0335 (7)	0.0836 (13)	0.0381 (8)	−0.0006 (8)	−0.0038 (6)	0.0134 (8)
N1	0.0319 (8)	0.0487 (10)	0.0329 (8)	−0.0029 (7)	0.0102 (6)	0.0036 (7)
C3	0.0230 (7)	0.0322 (9)	0.0288 (8)	−0.0031 (6)	0.0074 (6)	−0.0050 (6)
C1	0.0315 (8)	0.0325 (9)	0.0296 (8)	−0.0017 (7)	0.0112 (7)	−0.0012 (7)
C2	0.0268 (8)	0.0288 (9)	0.0268 (8)	−0.0006 (6)	0.0069 (6)	−0.0022 (6)
C7	0.0394 (9)	0.0377 (10)	0.0292 (9)	0.0014 (7)	0.0116 (7)	0.0045 (7)
C6	0.0373 (9)	0.0380 (10)	0.0268 (8)	0.0056 (7)	0.0050 (7)	0.0033 (7)
C5	0.0275 (8)	0.0311 (9)	0.0271 (8)	0.0003 (6)	0.0045 (6)	−0.0016 (7)
C4	0.0250 (8)	0.0327 (9)	0.0256 (8)	−0.0003 (6)	0.0054 (6)	0.0001 (6)
C8	0.0228 (7)	0.0328 (9)	0.0270 (8)	−0.0023 (6)	0.0035 (6)	−0.0034 (6)
C9	0.0211 (7)	0.0429 (10)	0.0293 (8)	0.0034 (7)	0.0037 (6)	−0.0020 (7)
C10	0.0232 (7)	0.0445 (11)	0.0298 (8)	0.0001 (7)	0.0051 (6)	−0.0034 (7)
C11	0.0290 (8)	0.0323 (9)	0.0268 (8)	−0.0007 (7)	0.0028 (6)	−0.0023 (7)
C12	0.0231 (7)	0.0381 (10)	0.0320 (8)	0.0036 (7)	0.0006 (6)	−0.0013 (7)
C13	0.0197 (7)	0.0416 (10)	0.0331 (8)	−0.0006 (7)	0.0054 (6)	−0.0041 (7)
C15	0.0293 (8)	0.0410 (10)	0.0296 (8)	0.0010 (7)	0.0016 (7)	0.0007 (8)

### Geometric parameters (Å, °)

**Table:**

Cl1—C15	1.803 (2)	C6—H6	0.9500
Cl2—C11	1.7373 (18)	C5—C4	1.365 (2)
O2—C3	1.3461 (19)	C5—C15	1.475 (2)
O2—N1	1.417 (2)	C4—H4	0.9500
O1—C15	1.186 (2)	C8—C9	1.400 (2)
N1—C1	1.320 (2)	C8—C13	1.401 (2)
C3—C2	1.382 (2)	C9—C10	1.379 (3)
C3—C8	1.452 (2)	C9—H9	0.9500
C1—C7	1.423 (3)	C10—C11	1.385 (2)
C1—C2	1.434 (2)	C10—H10	0.9500
C2—C4	1.420 (2)	C11—C12	1.384 (2)
C7—C6	1.356 (3)	C12—C13	1.383 (2)
C7—H7	0.9500	C12—H12	0.9500
C6—C5	1.446 (2)	C13—H13	0.9500
			
C3—O2—N1	111.15 (13)	C2—C4—H4	120.9
C1—N1—O2	104.15 (13)	C9—C8—C13	118.86 (16)
O2—C3—C2	108.11 (15)	C9—C8—C3	120.21 (15)
O2—C3—C8	116.92 (15)	C13—C8—C3	120.92 (15)
C2—C3—C8	134.97 (15)	C10—C9—C8	120.70 (16)
N1—C1—C7	126.85 (16)	C10—C9—H9	119.6
N1—C1—C2	112.17 (16)	C8—C9—H9	119.6
C7—C1—C2	120.98 (15)	C9—C10—C11	119.16 (16)
C3—C2—C4	135.77 (16)	C9—C10—H10	120.4
C3—C2—C1	104.42 (14)	C11—C10—H10	120.4
C4—C2—C1	119.77 (16)	C12—C11—C10	121.64 (16)
C6—C7—C1	117.82 (16)	C12—C11—Cl2	119.33 (14)
C6—C7—H7	121.1	C10—C11—Cl2	119.02 (13)
C1—C7—H7	121.1	C13—C12—C11	118.92 (15)
C7—C6—C5	121.57 (17)	C13—C12—H12	120.5
C7—C6—H6	119.2	C11—C12—H12	120.5
C5—C6—H6	119.2	C12—C13—C8	120.72 (15)
C4—C5—C6	121.66 (15)	C12—C13—H13	119.6
C4—C5—C15	122.78 (16)	C8—C13—H13	119.6
C6—C5—C15	115.56 (16)	O1—C15—C5	127.12 (18)
C5—C4—C2	118.18 (15)	O1—C15—Cl1	117.88 (15)
C5—C4—H4	120.9	C5—C15—Cl1	115.00 (13)

### Hydrogen-bond geometry (Å, °)

**Table:**

*D*—H···*A*	*D*—H	H···*A*	*D*···*A*	*D*—H···*A*
C4—H4···Cl1	0.95	2.58	3.015 (2)	108
C6—H6···O1^i^	0.95	2.53	3.378 (2)	149
C9—H9···Cl1^ii^	0.95	2.96	3.813 (2)	150
C13—H13···O2^iii^	0.95	2.69	3.492 (2)	142

Symmetry codes: (i) −*x*+1/2, −*y*+5/2, −*z*; (ii) −*x*+1/2, *y*+1/2, −*z*+1/2; (iii) −*x*+1, *y*, −*z*+1/2.
